# Biosorption of Cr(VI) by *Ceratocystis paradoxa* MSR2 Using Isotherm Modelling, Kinetic Study and Optimization of Batch Parameters Using Response Surface Methodology

**DOI:** 10.1371/journal.pone.0118999

**Published:** 2015-03-30

**Authors:** Melvin S. Samuel, M. E.A. Abigail, Chidambaram Ramalingam

**Affiliations:** School of Bioscience and Technology, VIT University, Vellore, Tamil Nadu, India; University of Copenhagen, DENMARK

## Abstract

This study is focused on the possible use of *Ceratocystis paradoxa* MSR2 native biomass for Cr(VI) biosorption. The influence of experimental parameters such as initial pH, temperature, biomass dosage, initial Cr(VI) concentration and contact time were optimized using batch systems as well as response surface methodology (RSM). Maximum Cr(VI) removal of 68.72% was achieved, at an optimal condition of biomass dosage 2g L^−1^, initial Cr(VI) concentration of 62.5 mg L^−1^ and contact time of 60 min. The closeness of the experimental and the predicted values exhibit the success of RSM. The biosorption mechanism of MSR2 biosorbent was well described by Langmuir isotherm and a pseudo second order kinetic model, with a high regression coefficient. The thermodynamic study also revealed the spontaneity and exothermic nature of the process. The surface characterization using FT-IR analysis revealed the involvement of amine, carbonyl and carboxyl groups in the biosorption process. Additionally, desorption efficiency of 92% was found with 0.1 M HNO_3_. The Cr(VI) removal efficiency, increased with increase in metal ion concentration, biomass concentration, temperature but with a decrease in pH. The size of the MSR2 biosorbent material was found to be 80 μm using particle size analyzer. Atomic force microscopy (AFM) visualizes the distribution of Cr(VI) on the biosorbent binding sites with alterations in the MSR2 surface structure. The SEM-EDAX analysis was also used to evaluate the binding characteristics of MSR2 strain with Cr(VI) metals. The mechanism of Cr(VI) removal of MSR2 biomass has also been proposed.

## Introduction

The swift industrialization has led to the enormous economic growth as well as serious irreversible environmental impact. These technological problems have been considered as one of the most substantial issues, especially in developing countries like India [1]. Unlike the existence of several heavy metals, release of chromium much beyond the permissible quantities was noticed in several countries. Chromium is considered as a toxic pollutant, mainly because of the existence in anionic and oxyanion forms. So, its contamination is being considered as one of the gravest environmental problems in the last few decades. Chromium is mainly released from industrial effluents through processes such as electroplating, leather tanning, nuclear power plant, textile industries, chromate preparation, refining processes, industrial dyes, pigments, film and photography, metal cleaning, galvanometric and electric industries [2].

Chromium exists in eleven valence states, ranging from −IV to +VI, among which Cr(III) and Cr(VI) are more stable in the environment. When compared to Cr(III), Cr(VI) is 100-fold times more toxic, mainly due to its high water solubility and mobile nature. The United States Environmental Protection Agency (USEPA) has set the maximum contaminant level (MCL) for Cr(VI) in domestic water supplies as 0.05 mg L^−1^, while total chromium content is regulated to be below 2 mg L^−1^ [3]. The toxicological effect of Cr(VI) originates from the action of its oxidizing property and also due to the formation of free radicals during the reduction of Cr(VI) to Cr(III) that occurs inside the cell. Cr(VI) is known for its neurotoxicity, genotoxicity, carcinogenicity and immunotoxicity [4]. The US EPA has listed chromium in the Class A Human Carcinogens list [5]. The currently available treatments for processing chromium-containing wastewaters *viz*. precipitation, ion exchange, reverse osmosis, evaporation and electro dialysis, are reported to exhibit reduced efficiency [6–12]. Nowadays industries have begun to seek alternative ways for treating chromium. Thus, there is a need to develop both economical and eco-friendly methods for Cr(VI) treatment.

Conventional treatment methods have disadvantages compared to the biological processes when it comes to its application at large scale. Biosorption is an emerging and smart technology, which involves sorption of dissolved substances by a biosorbent. [13,14]. It is a potential technique for the removal of heavy metals from solutions and recovery of precious metals. This technique could significantly reduce the cost, chemical usage and pollution compared to the other traditional Cr(VI) removal processes. Biosorption is a metabolic process, performed by both living and dead microbial cells. Although microorganisms such as bacteria and fungi were investigated massively, fungi were found to be more promising with excellent Cr(VI) removing capacity. This is mainly because (a) fungi are one such genera which can yield high amount of biomass (b) can survive in extreme environmental conditions and (c) highest percentage of cell wall material with outstanding metal binding properties. Diverse studies have also suggested that dead cells can be more effective for Cr(VI) removal than live cells. So, in the present study, we isolated a new indigenous soil fungal strain from a heavily polluted industrial effluent area and screened for its Cr(VI) removal capacity. The objectives of the present work were (a) to screen for a novel fungal biomass with high Cr(VI) removal capacity, (b) to study the effects of various experimental parameters *viz*. pH, temperature, biomass dosage, initial Cr(VI) concentration and contact time, (c) to find the rate of biosorption mechanism through isotherm and kinetic models, (d) to study the effect of temperature on biosorption through thermodynamic parameters and (e) to determine the effects of important experimental variables such as initial Cr(VI) concentration, contact time and biomass dosage on Cr(VI) percentage removal using a three-variable Box-Behnken Design (BBD).

## Materials and Methods

### Isolation and Biomass preparation

A fungus was isolated from a highly contaminated soil of an industrial effluent area in Vellore, Tamil Nadu, India and was designated as MSR2 strain. No specific permission was required for sample collection from the above mentioned site located at 12.9°N and 79.9°E. The culture was maintained on potato dextrose agar (PDA) media slants at 4°C throughout this study. For molecular identification, the fungal genomic DNA was isolated using Insta Gene TM matrix genomic DNA isolation kit and 18s rRNITS region was amplified using universal primers “ITS1”-“TCCGTAGGTGAACCTGCGG” and “ITS4”-“TCCTCCGCTTATTGATATGC”. The sequencing regions were submitted to the GenBank under the accession number KJ881375. The isolated fungi were identified by Yaazh Xenomics, Chennai, India. The fungi were cultured in the filamentous form under aerobic condition for 3 days in yeast extract, peptone glucose (YPG) media, which consists of yeast extract 3 g L^−1^, peptone 10 g L^−1^ and dextrose (a-D-glucose) 20 g L^−1^. The pH of the growth media was adjusted to 4.5 with 0.1 M HCl and incubated at 27°C. After three days of incubation, the biomass were collected and dried at 60°C temperature in oven for 24 h. The dried biomass was sieved through a 150-mesh sieve and used for biosorption experiments.

### Reagents and Instrumentation

A stock solution of chromium was prepared by dissolving a known amount of potassium dichromate K_2_Cr_2_O_7_ in deionised water (Sigma Aldrich Pvt. Ltd, India).

### Batch biosorption studies

Batch biosorption experiments were conducted in 250 ml Erlenmeyer flask containing 100 ml working solution with desired Cr(VI) concentration contacted with necessary MSR2 biomass and incubated for 24 h. After incubation, the samples were centrifuged at 5,000 rpm and the supernatant was analyzed for residual Cr(VI) concentration. For optimization studies, the experimental parameters such as pH (1.0–7.0), temperature (22–42°C), biosorbent dosage (1–3 g L^−1^), initial Cr(VI) concentration (25–125 mg L^−1^) and contact time (0–150 min) were varied. After incubation, the biomass were then separated by Whatmann no. 1 paper and the supernatant was analyzed for residual Cr(VI) metal concentration spectrophotometrically (UV 1800, Shimadzu, Japan) at 540 nm using diphenyl carbazide method.

The biosorption percentage of Cr(VI) ion was calculated as follows

$$\mathrm{Biosorption}(\%)=\frac{C_{0}-C_{f}}{C_{0}}\times 100$$ (1)

$$removal(\%)=\frac{C_{0}-C_{f}}{C_{0}}\times 100$$ (2)

The amount of Cr(VI) adsorbed by the MSR2 was calculated using the mass balance equation

$$q_{e}=\frac{Co-C_{f}}{M}\times V$$ (3)

Where q_e_ is the Cr(VI) ion uptake capacity (mg g^−1^); C_o_ and C_f_ are the initial and final Cr(VI) ion concentration (mg g^−1^); M is the dry weight of biosorbent (g) and V is the solution volume. Each experiment was performed in triplicates and the mean standard error was calculated.

### Biosorption isotherm modelling and uptake kinetics

The analysis of equilibrium data was performed using the mathematical methods for quantitative description of the obtained results. Liu and Liu (2008) stated that the equation parameters and the underlying thermodynamic assumptions of the isotherm models can predict the mechanism of the Cr(VI) uptake. Several biosorption isotherm models *viz*. Langmuir, Freundlich, Dubinin-Radushkevich (D-R), Temkin, Harkins-Jura, Hasley and BET isotherm were used in the study for fitting the experimental data (Table 1).

**Table 1:** Isotherm model regression constants for different experimental conditions.

Isotherms	
**Langmuir**	
q_max_ (mg/g)	72.46
B	0.022
R_L_	0.312
R^2^	0.991
**Freundlich**	
K_f_	1.05
n	1.08
R^2^	0.97
**Dubinin-Radushkevich (D-R)**	
E(KJ/mol)	10.31
q_max_(mg/g)	26.38
ß	0.70
R^2^	0.95
**Temkin**	
A (J/mol)	2.65
B (L/g)	0.115
R^2^	0.94
**Harkins-Jura**	
**A**	55.1
**B**	1.71
R^2^	0.82
Hasley	
n	1.19
R^2^	0.85
**BET**	
B	5.36
Q	3.30
R^2^	0.93

The Langmuir isotherm is used to analyze the adsorption capacity of adsorbent used and to predict the uptake of metal ions that occurs either on a homogeneous surface or heterogeneous surface [15], given by equation (4) and (5). The Freundlich model [16] applies to adsorption onto heterogeneous surfaces with a uniform energy distribution and reversible adsorption, given by equation (6) and (7)

$$q_{e}=\frac{q_{\max}bC_{eq}}{1+bC_{eq}}$$ (4)

The above mentioned equation is linearized as

$$\frac{1}{q_{e}}=\frac{1}{q_{\max}}+\frac{1}{q_{\max}b}\frac{1}{C_{eq}}$$ (5)

$$q_{e}=K_{f}C_{eq}{}^{1/n}$$ (6)

$$\log q_{e}=\log K_{f}+\frac{1}{n}\log C_{eq}$$ (7)

Where C_eq_ is the residual Cr(VI) concentration in solution, q_max_ is the maximum metal uptake (mg g^−1^), b the Langmuir equilibrium constant (L mg^−1^), K_f_ is the Freundlich constant (L g^−1^) and ‘n’ is the Freundlich exponent.

The D-R isotherm is generally used for distinguishing whether the adsorption is either physical or chemical adsorption of metal ions [17]. The parameter E can be correlated as:

$$E=\frac{1}{\sqrt{-2\beta }}$$ (8)

The D-R equation is given as

$$\ln q_{e}=\ln q_{\max}-\beta \varepsilon^{2}$$ (9)

$$\varepsilon =RT\ln(1+\frac{1}{C_{e}})$$ (10)

The Temkin isotherm model takes into account the adsorbent-adsorbate interactions [18] based on the assumption that the free energy of sorption is a function of the surface coverage. The non-linearized and linearized form of Temkin equation is expressed as

$$q_{e}=\frac{RT}{b_{T}}(\ln A_{T}C_{eq})$$ (11)

$$q_{e}=B_{T}\ln A_{T}+B_{T}\ln C_{eq}$$ (12)

Where b_T_ is the Temkin constant (J mol^−1^), A_T_ the temperature isotherm constant (L g^−1^), R (8.314 J mol^−1^ K^−1^) the universal gas constant and T the absolute temperature.

The Harkins-Jura isotherm explains the account for the multilayer adsorption and the existence of a heterogeneous pore distribution [19]. The linearized form of Harkins equation is given below

$$\frac{1}{q_{e}{}^{2}}=(\frac{B}{A})-(\frac{1}{A})\log C_{e}$$ (13)

The Hasley isotherm is suitable for multilayer adsorption and for fitting the experimental data to the heterogeneous nature of the adsorbent [20]. The non-linearized and linearized form of equation is given as

$$q_{e}={\left(\frac{K_{H}}{C_{e}}\right)}^{\frac{1}{\eta_{H}}}$$ (14)

$$\ln q_{e}=(\frac{1}{n})\ln k-(\frac{1}{n})\ln C_{e}$$ (15)

The BET isotherm model assumes that the Langmuir equation is applicable to each layer and a given layer may not be completely formed before the next layer forms.

$$q_{e}=\frac{q_{\max}BC_{eq}}{Cs-C_{eq}}+\left[1+(B-1)\frac{C_{eq}}{C_{s}}\right]$$ (16)

The linearized form of BET equation is given as

$$\frac{C}{(Cs-C){\left(q_{e}\right)}_{}}=\left[\frac{1}{BQ_{}}\right]+\left[\frac{B-1}{BQ_{}}\right]\left[\frac{C}{C_{s}}\right]$$ (17)

Where C_s_ is the saturation concentration of the solute.

On the other hand, the mechanism of biosorption process and the rate at which the processes take place were investigated by considering the processes of mass transfer and chemical reaction. For the study, several biosorption kinetic models such as fractional power, zero order, first order, pseudo-first order, second order [21], pseudo-second order, Elovich and intraparticle diffusion kinetics were used to fit the experimental data.

The fractional power model is a modified form of Freundlich model, expressed as

$$\ln q_{t}=\ln k+\nu \ln t$$ (18)

The pseudo-first-order equation also known as Lagergren equation and it is generally expressed as

$$\frac{dq}{dt}=K_{1}\left(q_{e}-q_{t}\right)$$ (19)

Where q_e_ and q_t_ are the adsorption capacities at equilibrium (mg g^−1^) at a time (t), and k_1_ the rate constant of pseudo-first order adsorption (min^−1^). The equation (19) can be re-arranged in the simplified form as

$$\log\left(q_{e}-q_{t}\right)=\log q_{eq}-\frac{K_{1}t}{2.303}$$ (20)

The pseudo-second order adsorption kinetic rate is expressed in equation (21)

$$\frac{dq}{dt}=K_{1}{\left(q_{e}-q_{t}\right)}^{2}$$ (21)

Where k_2_ is the rate constant of pseudo-second order adsorption (g mg^−1^ min^−1^). The equation can be linearized to

$$\frac{t}{q_{t}}=\frac{1}{K_{2}q_{e}^{2}{}_{}}+\frac{1}{q_{e}}t$$ (22)

The Elovich equation incorporates a logarithmic relation which states the diffusion of Cr(VI) as the rate controlling step and expressed as

$$\frac{dq}{dt}=a_{e}e_{t}^{-b_{e}q}$$ (23)

The linearized Elovich equation is given below

$$q_{t}=\frac{1}{b_{e}}(\ln a_{e}b_{e})+\frac{1}{b_{e}}\ln t$$ (24)

Where a_e_ is the initial adsorption rate (mg g^−1^) and b_e_ is the desorption constant related to surface coverage.

Intraparticle diffusion kinetics (Weber and Morris 1963) predict whether the adsorption is the rate limiting step or not at time‘t’. The mathematical equation of intra particle diffusion kinetics is given below

$$q_{t}=K_{id_{}^{}}t^{0.5}$$ (25)

Where q_t_ is the amount of Cr(VI) adsorbed on the surface, t is the contact time, K_id_ is the intra- particle diffusion coefficient. A linearized form of the equation is given as

$$\log q_{t}=\log k_{id}+0.5\;\log t$$ (26)

### Thermodynamic parameters

The temperature dependence of the biosorption process was analyzed with thermodynamic parameters *viz*. Gibbs free energy (ΔG°), enthalpy (ΔH°) and entropy (ΔS°) using the following equations

$$\Delta G{}^{\circ}=-RT\ln K_{c}$$ (27)

ΔG°=ΔH°−TΔS° (28)

$$K_{c}=\frac{q_{e}}{c_{f}}$$ (29)

Where K_c_ is the distribution coefficient for adsorption, R the gas constant (J mol^−1^K^−1^) and T the absolute temperature (K). Based on the Van’t Hoff plot of ln Kc verses T^−1^, the values of ΔH° and ΔS° were determined from the slope and intercept. The cell surfaces and the cell inclusions were visualized by atomic force microscopy (AFM). The samples without chromium were taken as controls.

### Particle size analyser

The size distribution of the fungal biomass was analyzed using Mile and Fraunhofer scattering particle size analyzer (Masterosizer 2000, Malvern Instruments Pvt Ltd., United Kingdom) of sizes from 0 to 2000 μm. For the analysis, reverse Fourier lens with convergent beam was utilized. The 21 CFR part 11 software which enables an operating mode that assists with ER/ES compliance with power of about 100 to 240 V and 50/60 Hz was taken for the study.

### SEM and EDAX analysis

The treated and untreated fungal biomass with Cr(VI) were analyzed using scanning electron microscope (Bruker). The surface morphology of the untreated and treated fungal biomass was carried out by Energy dispersive X-ray spectroscopy. The voltage was kept constant at 20 kV.

### Fourier transform infrared spectroscopy (FT-IR)

The FT-IR analyses within the range of 400–4000 cm^−1^ were recorded with an IR spectrometer (IR Affinity-1, Shimadzu, Japan) for Cr(VI) biosorption by MSR2 biomass at contact time of 0 and 60 min.

### Desorption experiments

The desorption experiments were repeated for 7 times to find the efficiency and reusability of MSR2 biosorbent. After the biosorption experiments, the adsorbed Cr(VI) on to the MSR2 biosorbent was separated from the solution by Whatmann no. 1 filter paper and then the separated MSR2 biosorbent was added in to solutions containing 0.1 M EDTA, 0.1 N HCl, and 0.1 N HNO_3_ and kept for 60 min at 27°C before determining the final Cr(VI) concentration. After each cycle of adsorption–desorption cycles, the MSR2 biosorbent was washed with distilled water, before successive cycles. Each new cycle of adsorption was carried out by supplementing 100 mg L^−1^ of Cr(VI). The desorption ratio of Cr(VI) ions from biosorbent was calculated from the amount of Cr(VI) ions adsorbed onto MSR2 biomass with the final Cr(VI) ion concentration in the desorption medium. Desorption ratio was calculated from the following equation

$$\text{Desorption ratio}=\frac{\text{amount of Cr(VI) ions desorbed to the desorption medium}}{\text{amount of Cr (VI) ions adsorbed onto the biosorbent}}\times 100$$ (30)

### Box-Behnken experimental design

Response surface methodology (RSM) is a collection of statistical and mathematical techniques that are used for refining the experiments and to process optimization. In this study, the RSM was performed to find out the percentage removal of Cr(VI) from the solution. The RSM was modelled statistically and designed by three parameter-factorial model. The highest, lowest and centre values of the parameters are given as +1, −1 and 0. Three independent factors *viz*. biosorbent dosage (A), initial Cr(VI) concentration (B) and contact time (C), were selected for the experimental design. Each experiment was repeated three times and the average mean value was used in optimization. The experimental sequence was randomized in order to minimize the effects of the uncontrolled factors.

The Cr(VI) removal percentage of fungal strain MSR2 was analyzed using second-order polynomial quadratic regression model equation as follows:

$$Y=\beta_{0}+\sum_{i=1}^{k}\beta_{i}x_{i}+\sum_{i=1}^{k}\beta_{\mathrm{ii}}x_{i}^{2}+\sum_{i-1}^{k-1}\sum_{j-1}^{k}\beta_{\mathrm{ij}}x_{i}x_{j}+\varepsilon$$ (31)

Where Y is the predicted response, x_i_, x_j_…, x_k_ were the input variables, x_i_^2^, x_j_^2^...., x_k_^2^ are given by square effects, ß_0_ is the intercept term, x_i_x_j_, x_j_x_k_ and x_i_x_k_ are the interaction effects, ß_i_ (i = 1, 2, … k) is the linear effect, ß_ii_ (1 = 1, 2, .., k) is the squared effect, ß_ij_ (j = 1, 2, .., k) is the interaction effect and є is the random error. Design-Expert 9.0 (Stat-Ease Inc, Minneapolis MN, USA) software was used for fitting the equations that is developed along with regression and graphical analysis for evaluating the statistical significance. The fittest and the accuracy of the model were evaluated by F value and the regression coefficient (R^2^). The optimal conditions for hexavalent chromium uptake by the fungal strain MSR2 was obtained by solving the regression equations and the 3D responses for the variable parameters using Design-Expert software version 9.0. The mathematical model obtained using RSM was validated by conducting experiments on given optimal conditions.

### Potentiometric titration

Potentiometric titration was performed with the help of a pH electrode, stirrer module and titration system. The pH electrode was calibrated with the buffer solution of pH 1.0, 4.0, 7.0 and 9.0. For each titration, 0.1 g of heat killed fungal biomass was added to 50 mL of 0.01 M KNO_3_ solution. Titration was carried out by drop wise addition of 0.06 ml of 0.1 M NaOH and the suspension was stirred at 200 rpm at room temperature. The experiments were carried out in the pH range from 3.0 to 10.

## Results and Discussion

### Isolation and identification of fungal strain MSR2 isolated from Ranipet

**Fig 1:**
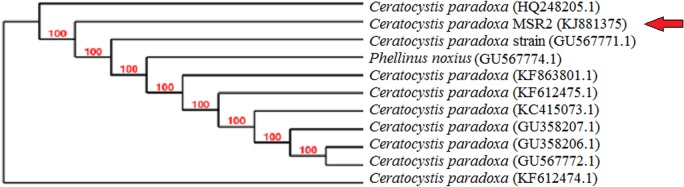
Neighbour- joining phylogenetic tree of 18s rRNA gene sequence of fungal strain MSR2 and most closely related species. The GenBank accession numbers for the corresponding sequences are given in brackets.

The fungal strain MSR2 was isolated from highly hexavalent chromium contaminated soil and was identified through molecular level characterization. The sequencing results were submitted in the NCBI GenBank database (Accession number: **KJ881375**). Using TREEVIEW software (1.6.6) phylogenetic tree was constructed for the strain MSR2 (Fig. 1). The strain MSR2 exhibited 99% homology with *Ceretocystis paradoxa* (Accession number: **HQ248205.1**) and *Ceretocystis paradoxa* (Accession number: **KC415073.1**) and identified as *Ceretocystis paradoxa* MSR2 strain. Moreover, to the best of our knowledge, this is the first report on the biosorption of Cr(VI) using fungi, *Ceretocystis paradoxa* MSR2 strain.

### Effect of pH and temperature

**Fig 2:**
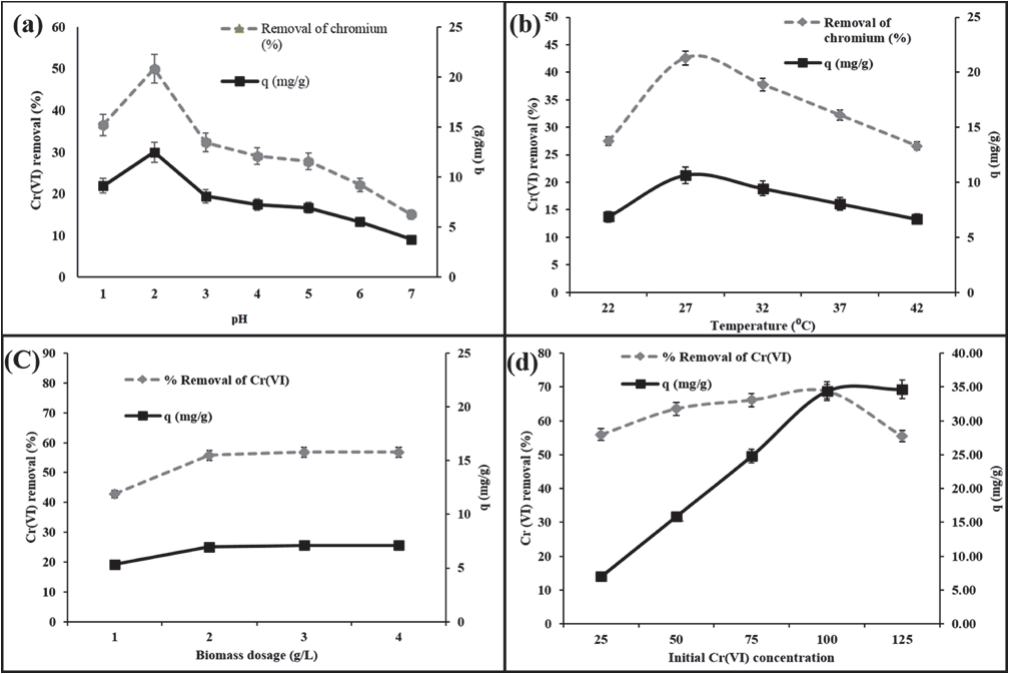
(a) Effect of pH (b) temperature (c) biomass dosage (d) initial Cr(VI) concentration on Cr(VI) biosorption (%) using *C*. *paradoxa* MSR2.

pH is an important parameter that affects the adsorption of metal ions as it changes the cell wall metal binding property. Therefore, to determine the effect of pH on Cr(VI) biosorption percentage on MSR2 biomass, the pH varied from 1.0 to 7.0. The Cr(VI) biosorption (%) was found to increase with a decrease in pH. The biosorption percentage decreased from 50.3% at pH 2.0 to 15.06% at pH 7.0. A similar trend was also noticed in case of uptake capacity (q_e_). Maximum Cr(VI) biosorption (%) was observed at pH 2.0. Therefore, further experiments were carried out at pH 2.0 (Fig. 2A). Similar results were also reported by Yang and Chen (2008) [23].

Similarly, a range of temperature (22°C–45°C) was chosen for the biosorption study. The Cr(VI) biosorption (%) decreased with an increase in temperature in case of both Cr(VI) percentage removal and uptake capacity. The biosorption efficiency of MSR2 decreased from 42.6% at 27°C to 14% at 45°C. The decrease in the biosorption percentage with rise in temperature might be due to desorption caused by an increase in the available thermal energy. Higher temperature induces higher mobility of the adsorbate causing desorption [24]. Optimum biosorption was achieved at 27°C and therefore further experiments were conducted in the optimized temperature (Fig. 2B).

### Effect of Biosorbent dosage

The biosorption of Cr(VI) was studied using different fungal biomass dosage (1–4 g L^−1^). The experimental results stated that the percentage of Cr(VI) biosorption and uptake capacity increased with increase in biomass dosage. This is due to the fact that the higher the amount of fungal adsorbent of the solution greater the availability of sites for binding to ions. The results obtained showed the maximum biosorption of 50% for an initial Cr(VI) concentration of 25 mg L^−1^ at a biomass dosage of 2 g L^−1^ which attained equilibrium at higher biosorbent dosage. The decrease in the MSR2 biosorption removal percentage at biosorbent dosages above 3 g L^−1^ might be due to partial aggregation of biomass, which has reduced the surface area available for biosorption. Therefore, the optimum biomass dosage was selected as 2 g L^−1^ for further experiment (Fig. 2C).

### Effect of initial concentration

The experiments were conducted at different Cr(VI) metal concentrations (25–125 mg L^−1^) at optimum temperature, pH, biosorbent dosage and contact time. The initial Cr(VI) concentration in the solution was found to be dependent on the equilibrium uptake. Maximum Cr(VI) percentage removal and uptake capacity were noticed at a concentration of 100 mg L^−1^, which was considered optimal (Fig. 2D). From the experimental results, it was evident that the Cr(VI) removal percentage has increased with increase in Cr(VI) concentration. This might be due to the fact that at higher Cr(VI) concentration an increase in driving force which overcomes all mass transfer resistance of metal ions between the aqueous and solid phase was attained, resulting in a collision between the metal ions and sorbents.

### Effects of contact time

The effect of contact time (0–150 min) for Cr(VI) biosorption was determined. The biosorption percentage of Cr(VI) increased (data not shown) with increase in contact time (68%) and reached equilibrium at 60 min after which no adsorption was noticed. In the initial stage, the Cr(VI) biosorption rate was faster as there were many free active binding sites left on the biosorbent surface. This shows that the biosorption is normally controlled by the diffusion process from bulk to the biosorbent surface, whereas in the later stage an attachment-controlled process could have happened likely due to less available biosorption sites. Hence, the contact time was optimized as 60 min for further studies.

### Isotherm modelling

The Langmuir isotherm model was actually developed to describe gas-solid phase adsorption on activated carbon and was later incorporated to analyze the biosorption of heavy metals onto the surface of the biomass [15]. This model assumes that the mode of adsorption is monolayer, where the adsorbed layer appears in one molecule in thickness. At the same time, it assumes that adsorption takes place only in a finite/fixed number of localized sites. The Langmuir adsorption isotherm derivation refers to the homogeneous adsorption mechanism where each molecule owns its enthalpy and activation energy at the same time. Also, this model does not permit transmigration of the adsorbate in the plane of the surface [24]. The parameters such as C_e_, q_e_ and C_e_/q_e_ were obtained from the Langmuir isotherm plot. To compare the model accuracy, the regression coefficients (R^2^) were calculated and listed in Table 1. The R^2^ value of Langmuir isotherm was greater than 0.99, which suggested the best fitting of the model with the biosorption data. The greater R^2^ value suggests the monolayer adsorption mode of Cr(VI) molecule on to the MSR2 biosorbent surface. The maximum monolayer biosorption capacity (q_max_) for MSR2 was about 72.46 mg g^−1^.

Freundlich isotherm is applicable for non-ideal reversible adsorption processes [16]. This model gives the information regarding multilayer adsorption with non-uniform distribution of heat which takes place on the heterogeneous surface [25]. At present, Freundlich isotherm is also applied for heterogeneous systems [26]. The parameters, q_e_ and R^2^, obtained from the Freundlich model, are given in Table 1. Freundlich constant n gives the favourability of the biosorption process. The regression coefficient (R^2^) was about only 0.97 suggesting the inappropriateness of the model to the current biosorption experiment. Dubinin–Radushkevich isotherm is used for the adsorption of vapour on to micropore solid surface followed by pore filling mechanism [17]. The D-R model is also applied to check whether the adsorption follows physic-sorption or chemi-sorption process [17]. In this study, the isotherm constant ß or B_DR_ was calculated as 0.0047. From the equilibrium data, the R^2^ value was found to be 0.76 and the intercept of the plots yielded a maximum uptake capacity, q_m_ of 2.6 x 10^–3^ mg g^−1^. Based on D-R isotherm the value of mean free energy (E), if it lies between 0–8 KJ mol^−1^ it denotes that the process follows physisorption where the value of E between 8–16 KJ mol^−1^ mentions chemisorption. From the obtained data, the value of E was found to be 10.314 KJ mol^−1^, which indicates that the experimental model follows chemisorption process.

Temkin isotherm exploits the information of adsorbent-adsorbate interaction and is based on the assumption that the free energy of sorption is a function of surface coverage, which does not take low and high level concentration into account [17–18]. A plot of q_e_ versus ln C_e_ enables the determination of the constants A and B. The constant B is related to the heat of adsorption while A is the equilibrium binding constant (L min^−1^) corresponding to maximum binding energy. The values of the constants A and B are tabulated in Table 1. Harkins-Jura isotherm equation is applicable for multilayer adsorption and explains the existence of heterogeneous pore distribution [19]. From the result, the value of R^2^ (0.86) was calculated and was found to be the lowest of all the isotherm equations studied earlier. So, it is evident that the adsorption of Cr(VI) did not follow a multilayer adsorption model. The Haley isotherm equation is also used for analyzing whether the mode of adsorption is multilayer. From the calculated results (Table 1), the regression value of the R^2^ (0.85) was found to be very low. Similar results were also obtained from the BET isotherm model. Therefore, the adsorption of Cr(VI) on to MSR2 biosorbent does not follow a multilayer mode of adsorption.

### Kinetic modelling

Biosorption of Cr(VI) on to *C*. *paradoxa* MSR2 strain and the rate limiting steps of kinetics *viz*. chemical reaction process, physical reaction process and mass transport, were examined by the values of the kinetic model constants. The parameters of the above mentioned models were calculated along with the relative correlation coefficient values (R^2^) are listed in the Table 2. The R^2^ values of fractional power, zero order, first order, pseudo first order and second order kinetics were calculated and were found to be lesser than 0.99. So, it was concluded that these models do not fit in the adsorption experiment. Also, the calculated ‘q_e_’ values of the pseudo-first order model contrasted the experimental ‘q_e_’ value. So, the model was not considered suitable for describing the biosorption kinetics of Cr(VI) by MSR2 strain. In contrast, the pseudo-second order model showed a high relative correlation coefficient value R^2^ greater than 0.998 (R^2^>0.99). Besides the calculated ‘q_e_’ values correlates with the experimental ‘q_e_’, which suggests that the biosorption follows the pseudo-second order kinetics at all the tested Cr(VI) concentrations. This implies that the biosorption of Cr(VI) onto fungal biosorbent MSR2 was *via* chemisorption process and the rate determining step is probably surface biosorption, which explains that the process occurs through valence forces such as sharing or exchange of electrons between adsorbent and Cr(VI) metal ions. The results obtained from the pseudo-first and pseudo-second order is in strong conformity with the results of other researchers [26]. Elovich model was also used to describe the second order kinetics. This model assumes that the solid surface is heterogeneous in nature and also has been accepted for defining the chemisorption process. The initial adsorption rate, a_e_ and desorption constant were calculated from the intercept and slope. The calculated values are illustrated in the Table 2. However, the experimental data did not give a good correlation with the presence adsorption studies.

**Table 2:** Kinetic parameters for the sorption of Cr(VI) on *Ceratocystis paradoxa* MSR2.

Kinetics	Parameters	Concentration (mg/L)				
	Experimental q_e_	25	50	75	100	125
		7.30	15.89	24.75	34.56	35.78
**Fractional power**	**K**	1.92	5.60	7.07	12.97	14.71
	**V**	0.405	0.315	0.302	0.243	0.221
	**R** ^**2**^	0.93	0.93	0.94	0.945	0.961
**Zero order**	**K**_**0**_	0.28	0.54	0.46	0.42	0.42
	**q**_**e**_	5.40	10.33	16.33	18.67	18.44
	**R** ^**2**^	0.962	0.960	0.970	0.971	0.971
**First order**	**K**_**1**_	0.100	0.102	0.057	0.055	0.057
	**q**_**e**_	5.39	10.33	17.63	20.69	21.22
	**R** ^**2**^	0.97	0.97	0.972	0.98	0.98
**Pseudo- first order**	**K**_**1p**_	0.100	0.102	0.057	0.055	0.057
	**q**_**e**_	5.42	10.32	17.37	20.65	21.23
	**R** ^**2**^	0.98	0.97	0.992	0.995	0.998
**Elovich**	**a**_**e**_	5.214	18.93	23.30	56.88	82.03
	**b**_**e**_	0.634	0.33	0.234	0.191	0.194
	**R** ^**2**^	0.99	0.99	0.963	0.99	0.990
**Second order**	**K**_**2**_	0.043	0.023	0.0091	0.007	0.008
	**q**_**e**_	5.40	10.41	28.32	29.41	42.91
	**R** ^**2**^	0.96	0.96	0.91	0.95	0.970
**Pseudo-second order**	**K**_**2p**_	1.02	1.23	0.29	0.34	0.35
	**q**_**e**_	7.29	15.89	25.31	34.96	36.49
	**R** ^**2**^	0.987	0.991	0.967	0.990	0.991
**Intraparticle diffusion**	**K**_**id**_	0.73	1.39	2.34	2.84	2.79
	**C**	2.50	6.73	8.03	13.62	15.34
	**R** ^**2**^	0.893	0.890	0.980	0.982	0.986

Intra particle diffusion is generally used for analyzing the diffusion of the particle. When the diffusion mechanism could not be explained by pseudo-second order and Elovich kinetic model, the intraparticle diffusion model developed by Weber and Morris (1963) come into play. According to this model, the plot q_t_ vs t^1/2^ should be linear and also the line should pass through the centre or the origin if intra-particle diffusion is the rate-controlling step. But if the line did not pass through the origin, then the other kinetic models may have contributed to the rate of adsorption [22].

In the present study, the intra particle diffusion plot was obtained between q_t_ vs t^1/2^ at different concentration and the K_i_ values were obtained from the slope. The correlation coefficients (R^2^) for the intraparticle diffusion model were found to be above 0.99 at all the tested concentrations (Table 2). From the Table 2, all the curves had the same features with an initial curve followed by a linear portion and later a plateau. The curved portion attributes to the boundary layer sorption while the linear portion of the intraparticle diffusion as well as the plateau to equilibrium. From the experimental result, it was evident that the straight line does not pass through the origin, which confirms that intra-particle diffusion is not the rate limiting step.

### Thermodynamic study

Thermodynamic parameters such as free energy (ΔG°), enthalpy (ΔH°) and entropy (ΔS°) were also studied. The parameter free energy (ΔG°) was calculated to be negative for the biosorption of chromium at different temperatures, 22, 27, 32, 37 and 42°C, respectively. The negative ΔG° values represent that the adsorption undergoes thermodynamically feasible reaction and also spontaneous in nature. The negative ΔH° value (−133.19 KJ mol^−1^) shows that the nature of adsorption is exothermic. Additionally, the obtained ΔH° value (43.1 KJ mol^−1^) showed that the adsorption of Cr(VI) onto the biomass *C*. *paradoxa* MSR2 follows chemisorption (Table 3). The results obtained from D-R were also in strong conformity with the results of thermodynamics. The negative ΔS° value (−43.4 J mol^−1^ K^−1^) suggests a decrease in the randomness during biosorption of Cr(VI) on to *C*. *paradoxa* MSR2. Further, the size of MSR2 biomass was found to be 80 μm size with the help of particle size analyser.

**Table 3:** Thermodynamics parameters for the sorption of Cr(VI) on *Ceratocystis paradoxa* MSR.

Temperature (K)	ΔG° (KJ mol^−1^)	ΔH° (KJ mol^−1^)	ΔS° (J mol^−1^ K^−1^)
**295**	−3.412		
**300**	−4.256	− 43.1	− 133.19
**305**	−5.123		
**310**	−5.782		
**315**	−7.162		

### Box-Behnken experimental design

The parameters such as adsorbent dosage, contact time and initial hexavalent chromium concentration were chosen for the Box-Behnken experimental design (Table 4 and Table 5).

**Table 4:** The level of variables selected for RSM design.

Factors and its codes	Level of factors		
	−1	0	+1
Biosorbent dosage (g L^−1^)	1.0	2.0	3.0
Initial Cr(VI) concentration (mg L^−1^)	25.0	62.50	100.0
Contact time (min)	15.0	37.50	60.0

**Table 5:** Box-Behnken Design matrix for three factors along with observed response for Cr(VI) biosorption by MSR2 biosorbent.

Run	A-Biomass dosage g L^−1^	B-Initial Cr(VI) concentration mg L^−1^	C-Contact time min	R1-Cr(VI) percentage removal (%)
**1**	1.000	1.000	0.000	39.78
**2**	0.000	0.000	0.000	35.78
**3**	0.000	0.000	0.000	35.78
**4**	−1.000	1.000	0.000	30.78
**5**	0.000	−1.000	−1.000	8.78
**6**	−1.000	−1.000	0.000	22.80
**7**	0.000	1.000	1.000	68.72
**8**	1.000	0.000	1.000	56.80
**9**	1.000	−1.000	0.000	32.89
**10**	1.000	0.000	−1.000	9.89
**11**	0.000	−1.000	1.000	55.80
**12**	0.000	0.000	0.000	35.78
**13**	−1.000	0.000	1.000	54.78
**14**	−1.000	0.000	−1.000	7.89
**15**	0.000	1.000	−1.000	11.24
**16**	0.000	0.000	0.000	35.78
**17**	0.000	0.000	0.000	35.78

In order, to find out the combined effect of all the above mentioned factors, experiments were performed in different combination of parameters using experimental design. Table 6 depicts the results of three factor Box Behnken design. The variance analysis for Cr(VI) removal of the BBD model is tabulated in Table 7. The model F-value of 72.56 implies that the model is significant. There was only 0.01% chance that F-value was out of design due to noise. The values of “Probability > F” less than 0.050 indicated that the model terms *viz*. A, B, C, AB, BC, B^2^ and C^2^ were significant. The Cr(VI) removal by variance analysis using BBD model is given in Table 7.

**Table 6:** ANOVA analysis for response surface second order model in relation to Cr(VI) biosorption.

Source	Sum of Squares	df	Mean Square	F Value	p-value Prob >F	
Model	5192.95	9	576.99	94.52	< 0.0001	significant
A-A-Biomass dosage	66.76	1	66.76	10.94	0.0130	
B-B-Initial Cr(VI) concentration	114.38	1	114.38	18.74	0.0034	
C-C-Contact time	4915.36	1	4915.36	805.20	< 0.0001	
AB	0.30	1	0.30	0.049	0.8317	
AC	1.000E-004	1	1.000E-004	1.638E-005	0.9969	
BC	27.35	1	27.35	4.48	0.0721	
A^2	67.58	1	67.58	11.07	0.0126	
B^2	0.19	1	0.19	0.031	0.8657	
C^2	1.35	1	1.35	0.22	0.6525	
Residual	42.73	7	6.10			
Lack of Fit	42.73	3	14.24			
Pure Error	0.000	4	0.000			
Cor Total	5235.68	16				

**Table 7:** Statistical analysis for the removal of hexavalent chromium by response surface model.

**Std. Dev.**	2.47	**R-Squared**	0.9918
**Mean**	34.06	**Adj R-Squared**	0.9813
**C.V. %**	7.25	**Pred R-Squared**	0.8694
**PRESS**	683.71	**Adeq Precision**	33.061

Where A is the biomass, B is the initial Cr(VI) concentration, C is the contact time and Y is the chromium removal (%) of MSR2 strain. The coefficient values of A, B and C are related to the effects of the three factors on the response Y.

**Fig 3:**
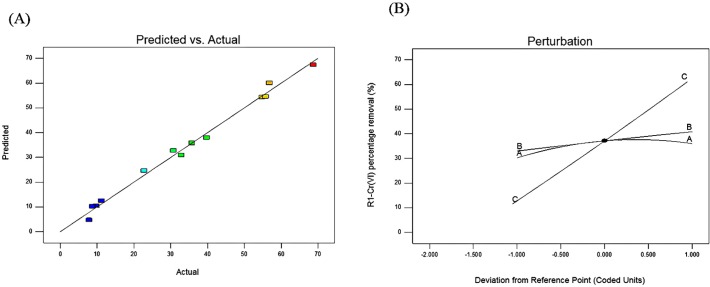
Actual versus predicted values for Cr(VI) percentage removal; The perturbation plot of factors biomass dosage (A), initial Cr(VI) concentration (B) and contact time (C).

Additionally, the statistical analysis with standard deviation and mean are listed in Table 7. The “Predicted R^2^” 0.8694 was found to be in reasonable agreement with “Adjusted R^2^” of 0.9813. It was also noticed that the “Adequate Precision” measured the signal to noise ratio. A ratio greater than 4 was desirable. The ratio of 33.061 indicated an adequate signal. Therefore, the model could be used to navigate the design space. The experimental data versus predicted value for Cr(VI) removal (%) is shown in Fig. 3A. It can be seen that most of the experimental values lie close to the straight line, which indicates the experimental values correlates with the predicted value. The results of the plot showed that increase in biomass concentration and contact time increased with the Cr(VI) removal percentage. However, the values of each parameter are different from others. Perturbation plot of three independent factors is shown in Fig. 3B. In this response design, the perturbation plot for each factor changes or moves from the reference point. In the model, the center point, factors C (contact time) shows a high effect on response as it changes from the reference point. Higher the contact time, the greater the amount of Cr(VI) ion removal. Factor A and B has the same effect as factor A.

The tested variables are presented for identifying the variables and their interaction *via* three dimensional response surface. The response surface explains the interaction between biomass dosage and initial concentration of Cr(VI) while the contact time was kept constant at 60 min. The biosorption of Cr(VI) increased with an increase in the biomass concentration 2.0 g L^−1^ and declines further with increase in biomass dosage. Further, the effect of interface between initial Cr(VI) concentration and contact time is also illustrated.

**Fig 4:**
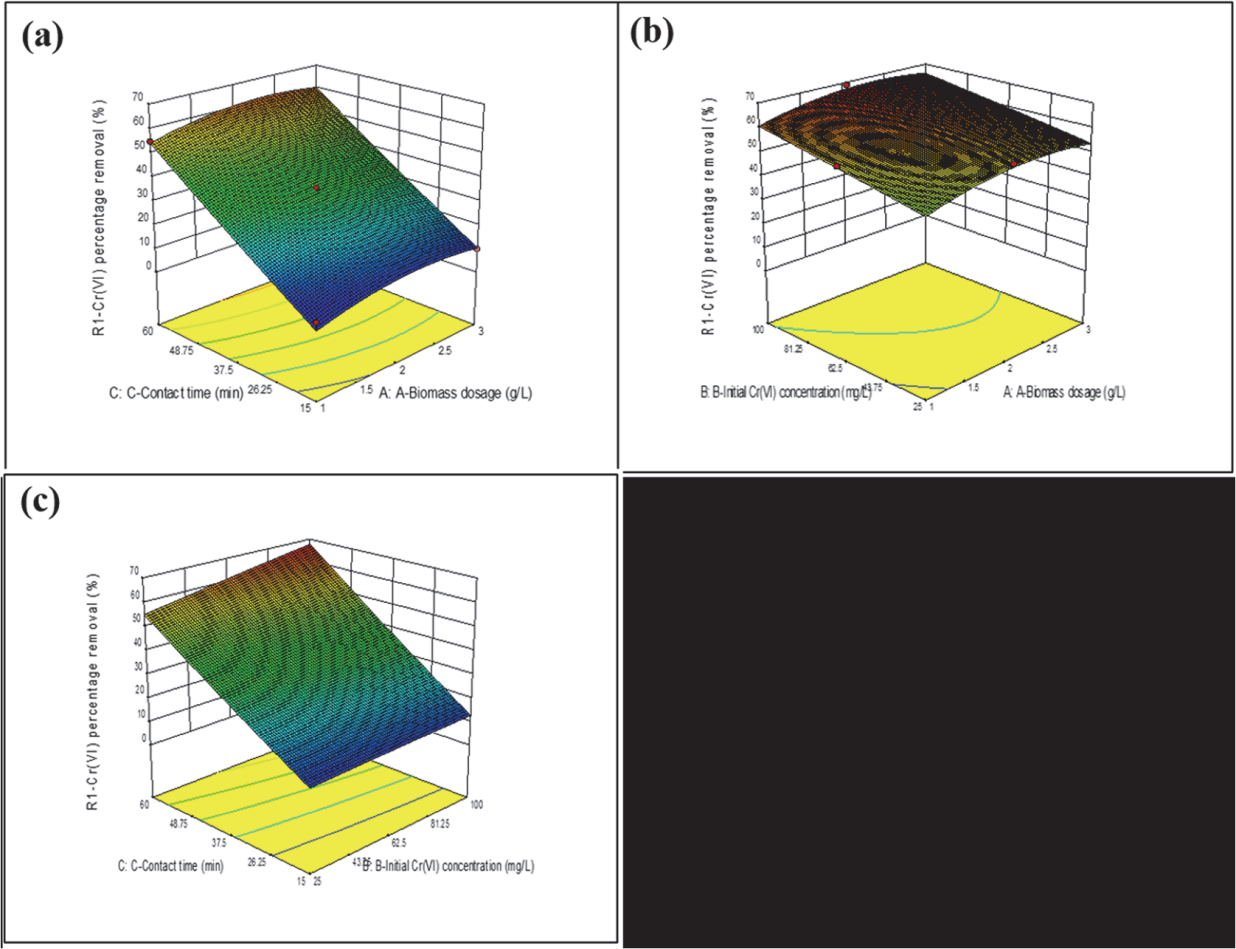
The 3D response surface plots showing the effects of interactions (a) biosorbent dosage on Cr(VI) removal; (b) initial Cr(VI) concentration on percent removal of Cr(VI); and (c) contact time on the percent of Cr(VI) removal. Note: A: biosorbent dosage (mg g^−1^); B: Cr(VI) concentration (mg L^−1^); C: contact time (min).

From Fig. 4A, B, C, maximum Cr(VI) removal of about 72.46% was achieved at a biomass dosage of 2 g L^−1^ and initial concentration of 62.5, while the contact time was 60 min. Additionally, the RSM optimization results were in accord with the optimization results. Table 8 compares the biosorption capacity of MSR2 biomass with the other fungal biomass. The highest Cr(VI) uptake was noted in the present study, which exhibits the efficiency of the MSR2 biosorbent for Cr(VI) removal.

**Table 8:** Comparison of other fungal biosorbents from literature with the present work.

Name of fungi	Sorption capacity (mg/g)	Reference
*Aspergillus niger*	30.1	[29]
*Aspergillus flavus*	0.335	[30]
*Coriolus versicolor*	44.25	[31]
*Lentinus sajor-caju* (free)	23.32	[32]
*Mucor hiemali*	53.5	[33]
*Penicillium purpurogenum*	40	[34]
*Rhizopus arrhizus*	23.88	[35]
*Saccharomyces cerevisiae*	32.6	[36]
*Penicillium purpurogenum*	40	[34]
***Ceratocystis paradoxa* MSR2**	**72.46**	**Present study**

### Morphological details of biosorbent adsorption

**Fig 5:**
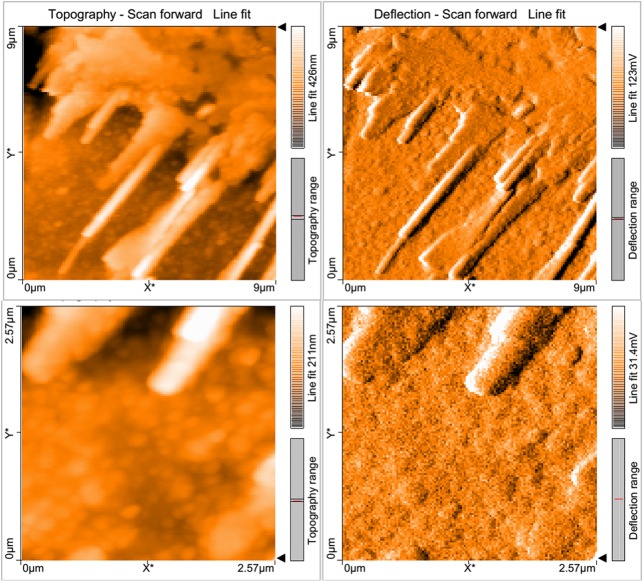
AFM images of MSR2 after biosorption: (a) scanning range 9μm and (b) scanning range 2.5μm.

**Fig 6:**
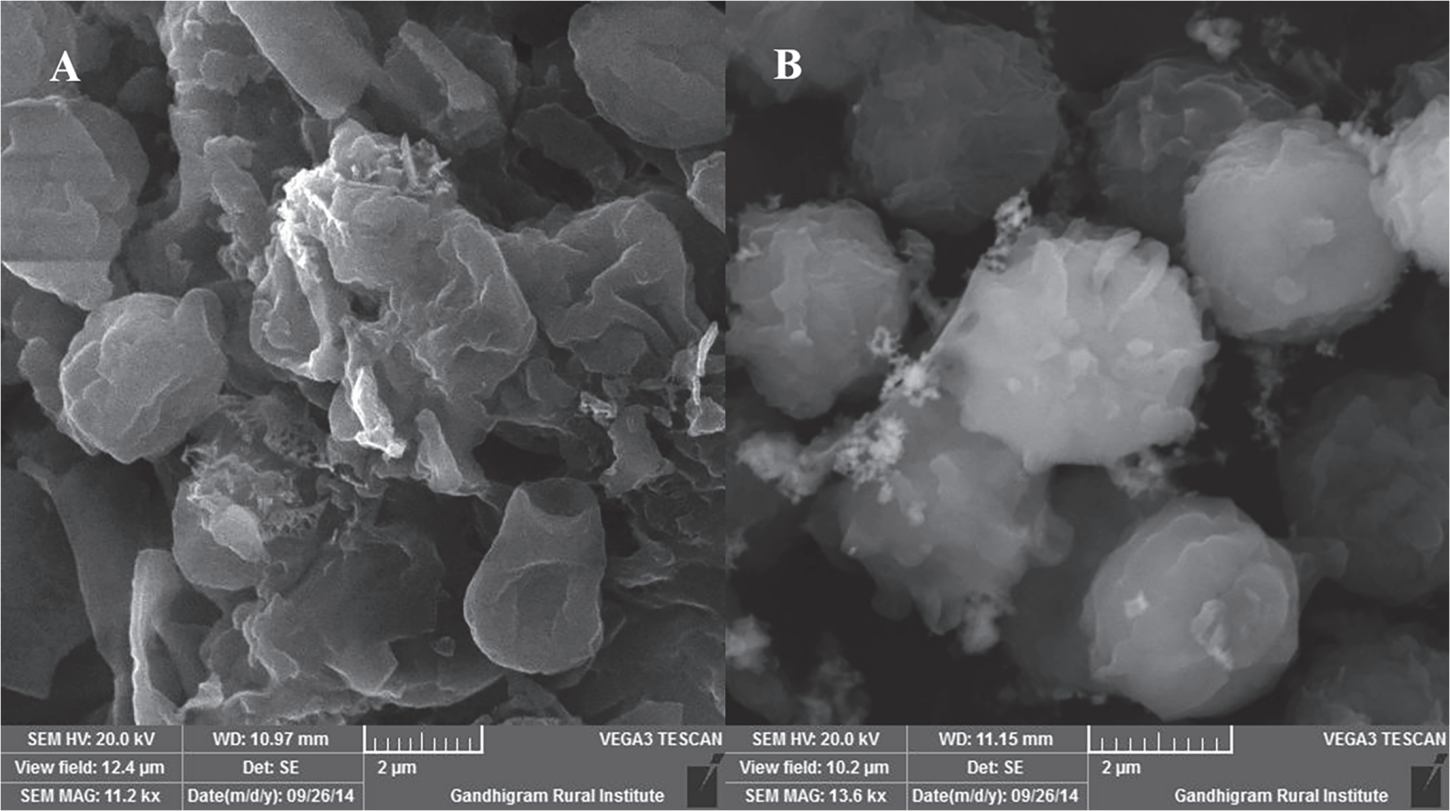
SEM analysis of MSR2 before (a) and (b) after Cr(VI) biosorption.

The adsorption on Cr(VI) onto the MSR2 biosorbent was visualized under AFM for the analysis of the morphological differences (Fig. 5A and B). The adsorption of Cr(VI) onto the fungal MSR2 could have altered the biosorbent configuration and has also created a more favorable condition for capturing Cr(VI). From Fig. 6, it is evident that a larger amount of Cr(VI) is adsorbed onto the MSR2 strain with macromolecule substance concentrate and results in the formation of a reticular bulging at the tip. Due to the acidic conditions, the intracellular amino groups ionizes and forms a positively charged surface for the adsorption of negative chromic ions onto them.

### Scanning electron microscopy

The SEM images of the untreated biomass displays a smooth surface whereas the treated biomass was found to be irregular, rough, clumps around and exhibited folds (Fig. 6).

### EDAX spectroscopy

**Fig 7:**
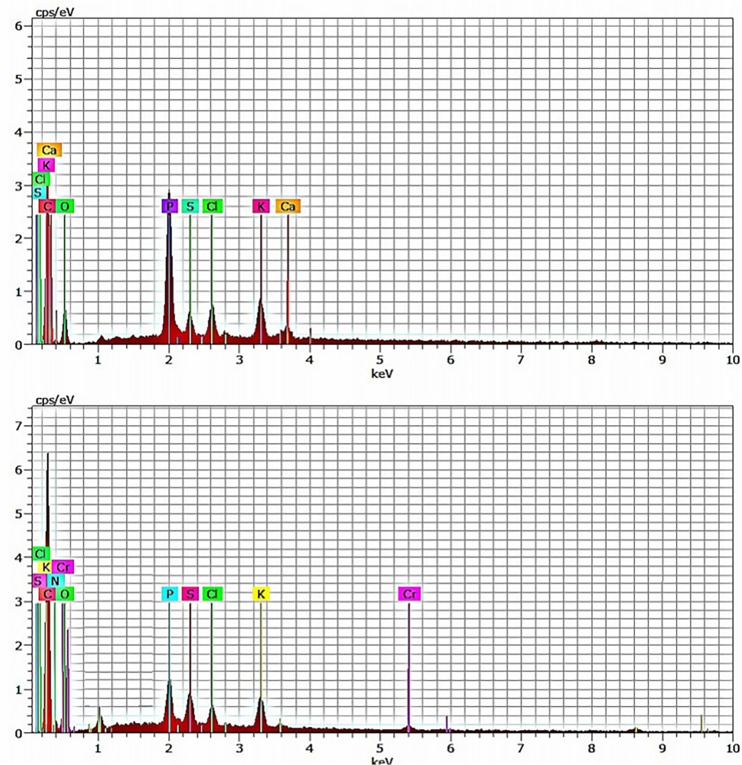
EDAX analysis of MSR2 before (a) and (b) after Cr(VI) biosorption.

The EDAX spectra for the treated fungal biomass confirms the presence of chromium ions on the sorbent layer. The decrease in calcium ion along with an increase in oxygen atom concentration were noticed in Cr(VI) treated sample (Fig. 7). Therefore, the results of SEM analysis were found to be in accordance with the equilibrium isotherm results.

### FT-IR spectrum of *C*. *paradoxa* MSR2

**Fig 8:**
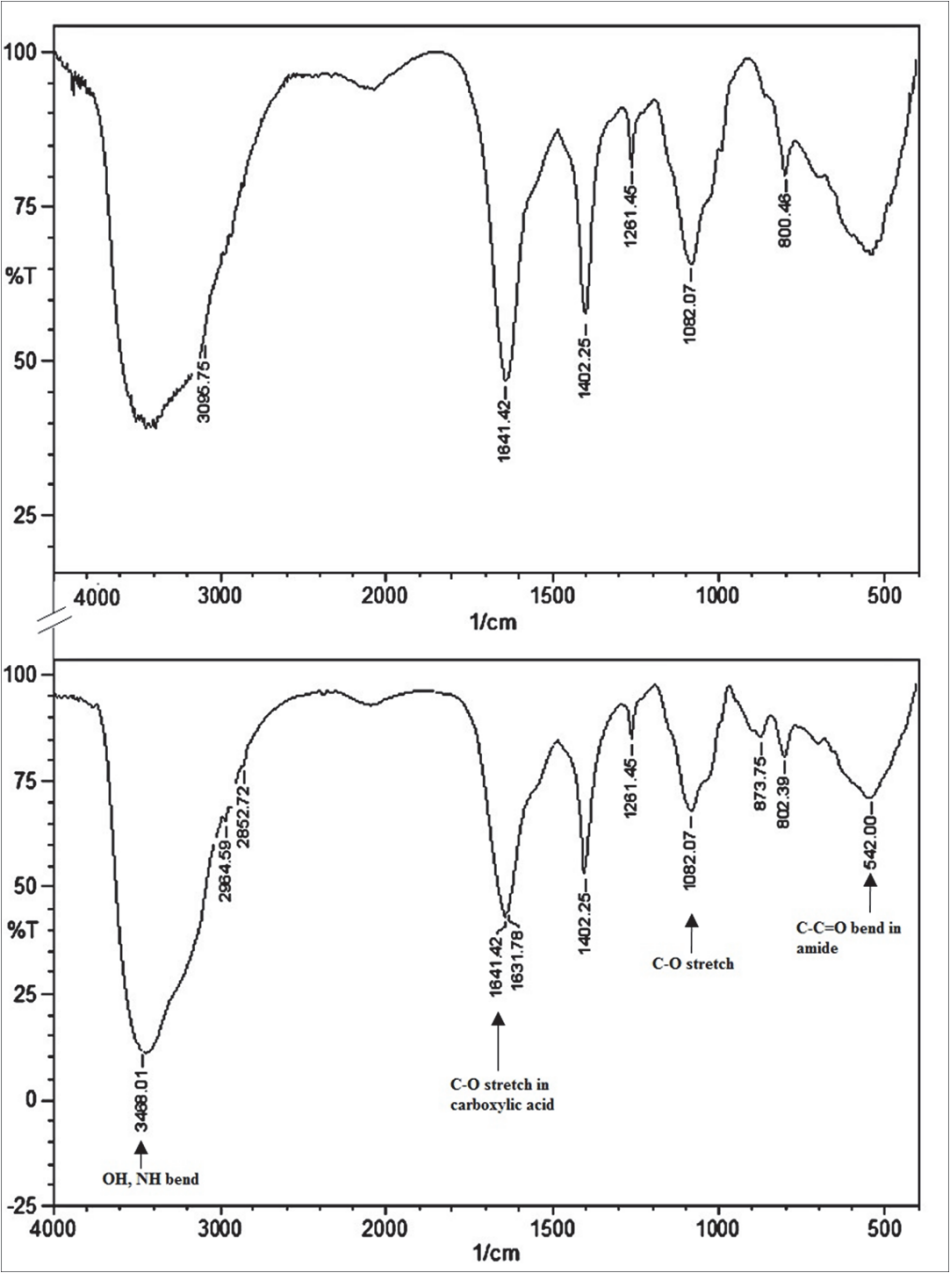
FTIR spectrum of MSR2 before (a) and (b) after Cr(VI) biosorption.

Fig. 8 presents the IR maximum of absorption for different functional groups before and after Cr(VI) sorption. The FT-IR spectrum of MSR2 strain showed the presence of the amino group, carboxylic group, carbonyl group and hydroxyl group. The presence of carboxylic acid was confirmed by the presence of −OH group along with carbonyl group. The amino (NH-), hydroxyl and carbonyl group describe the presence of amino acid in the biosorbent. The functional group such as −OH, −NH, −CO shows long stretching vibrations, which denotes a shift of functional groups to a certain extent in the MSR2 biosorbent. Similar results were also reported by other researchers [37–38].

### Desorption Studies

Desorption and regeneration studies using MSR2 biomass showed the possibility for regeneration and recovery of Cr(VI). Chemisorption or ion exchange was found to be the main mechanism for Cr(VI) attachment to the MSR2 biosorbent. So, the physical adsorption had played a minimal role in the process. The result of desorption studies of Cr(VI) in a batch system showed that 0.1 M HNO_3_ (92%) was more efficient in Cr(VI) desorption than the other solvents tested. It was evident from the results that with the concentration of the solvents were proportional to the desorption rate at the initial stage after which it reaches equilibrium. A gradual decrease in Cr(VI) sorption with an increase in the number of cycles was also noticed. Similar results were also obtained by Anayurt et al. [39].

### Mechanism of removal of Cr(VI) by dead fungal biomass

Earlier reports have claimed the removal of Cr(VI) from aqueous solution *via* adsorption anionic chromate mechanism where the anionic chromate binds with positively charged groups of the dead fungal biomass. In our study, we suggest that the total chromium analysis at the designated contact time demonstrates reduction of Cr(VI) to Cr(III) and then gets completely removed from the solution. Further, the desorption results indicated that most of chromium bound on the biomass might be Cr(III), mainly due to the principle of “redox reaction”. The results of the mechanistic study are in correlation with the results observed in other studies [27, 40].

The Cr(VI) present in the solution can be removed by two mechanisms (MI and MII) by the dead fungal biomass. In M1, the Cr(VI) readily gets reduced to Cr(III) in the aqueous solution by the fungal biomass whereas MII involves three steps. Firstly, the Cr(VI) binds to the positively charged groups such as amine present in fungal cell wall. Secondly, the adjacent functional groups having lower reduction potential converts Cr(VI) to Cr(III) and finally the reduced Cr(III) is released in to the aqueous solution by electronic repulsion between the positively charged groups and the cationic Cr(III) ion. In MII mechanism, the proton plays a vital role where the solution pH increases during each step of Cr(VI) removal by the dead fungal biomass. Thereby, it is necessary to supplement protons for enhancing the removal percentage of Cr(VI) by the dead fungal biomass. Similar results were also reported by other researchers [28, 41]. It was evident that the solution pH is the most important parameter in Cr(VI) removal using dead fungal biomass. Based on the FT-IR results, the shifts or changes of the peaks would indicate interactions between the metal with functional groups on the solid surface as a result of biosorption or a chemical reaction. As a result, the decrease in the intensities of these peaks indicated the oxidation of fungal biomass in the event of Cr(VI) reaction. However, the whole region of different functional group bands on the fungal biomass surface is involved in the biosorption of Cr(VI).

## Conclusion

In the quest of exploring new, efficient, cost effective and regenerative biosorbent, we report a new *Certocystis paradoxa* MSR2 biomass, as an ideal biosorbent for Cr(VI) removal. The current experiment on the study of Cr(VI) biosorption onto MSR2 depended on solution pH, initial Cr(VI) concentration and temperature. The isotherm data was best described by the Langmuir isotherm. Kinetic data agreed well with the pseudo-second order model. The thermodynamic parameters revealed the nature of the Cr(VI) biosorption onto MSR2 strain. The characterization of the biosorbent using FTIR spectrum revealed the role of functional groups such as hydroxyl, amino, carboxylic and carbonyl groups in Cr(VI) biosorption by MSR2 strain. Desorption studies using HNO_3_ (0.1 M) showed higher Cr(VI) desorption efficiency (>95%). From the study, we also noticed the strong conformity of experimental optimization values with the predicted RSM model values.
